# Supplementary material for: Patterns of Intron Gain and Loss in Fungi
Source: PLoS Biol. 2004 Nov 30;2(12):e422. doi: 10.1371/journal.pbio.0020422 (PMC532390; doi:10.1371/journal.pbio.0020422)
Supplement: Table S1 — Also available at http://genes.mit.edu/NielsenEtAl/. (4.3 MB ZIP). [file pbio.0020422.st001.zip › NielsenEtAl/html/487.html]

AN8032.1.NCU01177.1.MG08631.1.FG06786.1


```
 CLUSTAL W (1.82) Multiple Sequence Alignments - Introns Inserted


Sequence 1: NCU01177.1	338 aa
Sequence 2: MG08631.1	335 aa
Sequence 3: FG06786.1	348 aa
Sequence 4: AN8032.1	314 aa
Alignment Length: 383 aa
Number Identitical Residues: 145 aa
Alignment Score (without introns) 7061


MG08631.1 	M0KPADMTEKR-ALVTGATGLLGREVAKAFQ---------LKHGWT--VTGTAFSRADGV
NCU01177.1	M~SPVNNNQTKTALVTGATGLLGRQVVRAFQGLSVPTEVSSKAGWTWEVKGTGFSRADGV
FG06786.1 	-~-----MTDRTVLVTGATGLLGREVSAAFG----------LKNWE--VKGTGFSRADGI
AN8032.1  	-~------MATTVLVTGATGLLGRQVFDKFK----------RSGCL--TVGQGFSRANPP
          	           :.***********:*   *             .    . * .****:  

MG08631.1 	STLRADLANEAEVEKVLDQVK2PHVVVHC1AANRFPDKVDQDPEGTRRLNVAATASLARL
NCU01177.1	NVLKVDLEKAEEVERVLGDVS2PQVLVHC~AANRFPDKVDADPEGTRLLNVEATRTLARA
FG06786.1 	STFKVDLGNENEVSTFLDETK2PQVIVHC1AAQRFPDKVDKDPEGARALNVAASKALAKL
AN8032.1  	TILKSDLEKPDDIKGLLDEAK2PQIVIHC1AANRSPDLCDKDPERARRVNVDATRTLAEL
          	. :: ** :  ::. .*.:.. *::::** **:* **  * *** :* :** *: :**. 

MG08631.1 	CADRDVALIYISTDYVFSGRPGEAPYAAGAATGPTNLYGQTKLDGEKAVLEEMAAK---G
NCU01177.1	CASRGILLIYISTDYVFSGKPGEAPYEADAATGPTNLYGVTKLDGEKAVIEEFSKASKEG
FG06786.1 	AAERDTFVIYISTDYVFPGVPGDAPYEADAEPRPTNLYGQTKLDGERAVLETFKEAGKEG
AN8032.1  	TAERNILLVYISTDYVFPGVEGEAPYEADAPTKPPNLYGELKRDGEQVVLEATKKS---G
          	 *.*.  ::********.*  *:*** *.* . *.****  * ***:.*:*        *

MG08631.1 	RGVVMRVPVLYGHAETPG--ESAVNVLMDAVWKSQEAETKIKMDHWAIRYPTNTEDVGRV
NCU01177.1	LGVVMRVPVLYGKTEEGRNEESAVNVLLDAVLKAQEGK-KVKMDHWALRFPANTEDVGRV
FG06786.1 	LGVVLRVPVLYGNAETPS--ESAVNVLMDALWKAQTQGAQISMDHWAIRYPTNTEDIGRV
AN8032.1  	MGIILRVPVLYGTANENS--ESAVNTLVDSVWKAQDENAGIKMDDWAQRYPTNTEDVARV
          	 *:::******* ::     *****.*:*:: *:*   : :.**.** *:*:****:.**

MG08631.1 	CAG1SKGWLTMVFAFLLQD~VAEKYVDAQDAK--SLPKVLQFSSEDKLTKYEICQVFGEI
NCU01177.1	CR-~--------------D1VAVKYLSTSEAERTSLPRILQFSGEEQYTKYEMCQLFAEI
FG06786.1 	CH-~--------------D1ISVKYLDTPSNNRASLPNILQFSSEDRMTKYEIVALFGEI
AN8032.1  	LR-~--------------D~IVIKYLKE-RGQIMKLPKILQFSSEDKMTKYEMCEKFAEI
          	                  * :  **:.    :  .**.:****.*:: ****:   *.**

MG08631.1 	MGLSIEGIEANTQGNDPNASVQRPYDCHLSTKELKDLGIDVSTQDFG-~---------DW
NCU01177.1	LGVPVDNIEANTEGNDPNASVQRPYDCHLSTKALKDLGIDVSTQDFA-~---------AW
FG06786.1 	MGLSTEGITPNTEGNDRNASVQRPYDCHLSTKALKDLGIDVSTCDFKD1ETKWFFEWSGW
AN8032.1  	LGVSLKGMVRNKEGNDPNASVQRPYDTHLSTKVLKDLGIDVQTVDFV-~---------DW
          	:*:. ..:  *.:*** ********* ***** ********.* **             *

MG08631.1 	W~RREVKA~FRK------~-------------
NCU01177.1	W~RRELRA~FRH------~-------------
FG06786.1 	F~LMVEKG0VLKVMLWPL0FEMGTFLLSSNSC
AN8032.1  	W2RKHLEA~QKK------~-------------
          	:     ..   :
```
